# Kinetics of Enzymatic Synthesis of Cyanidin-3-Glucoside Lauryl Ester and Its Physicochemical Property and Proliferative Effect on Intestinal Probiotics

**DOI:** 10.3390/biology9080205

**Published:** 2020-08-04

**Authors:** Xi Yang, Hanju Sun, Lijun Tu, Yuan Jin, Zuoyong Zhang, Muwen Wang, Shuyun Liu, Ying Wang, Shudong He

**Affiliations:** 1 School of Food and Biological Engineering, Hefei University of Technology, Hefei 230009, China; yangxi170420@163.com (X.Y.); lijuntu@mail.hfut.edu.cn (L.T.); 18255333092@163.com (Y.J.); 18856311272@163.com (Z.Z.); 18742514809@163.com (M.W.); sallyliushuyun@163.com (S.L.); lxn170420@126.com (Y.W.); 2 Engineering Center of Ministry of Agricultural Products Processing Education, Hefei University of Technology, Hefei 230009, China

**Keywords:** Cyanidin-3-glucoside, kinetic model, enzymatic synthesis, physicochemical property, proliferative effect, intestinal probiotics

## Abstract

The interest in anthocyanins used in food, cosmetic, and pharmaceutical industries has increased the research in order to improve their stability while maintaining bioactivity. In this work, cyanidin-3-glucoside lauryl ester (Cy3glc-C12) was enzymatically synthesized, using Novozym 435 as a catalyst, as well as to obtain a kinetic model for the bioprocess. Its liposolubility, UV–VIS absorbance property, thermostability, and potential proliferative effect on intestinal probiotics were also studied. The maximum conversion yield (68.7 ± 2.1%) was obtained with a molar ratio (substrate:donor) of 1:56, 435 16.5 g/L Novozym, temperature of 56 °C, and a time of 28 h via the acylation occurred at 6′′-OH position of the glucoside. The kinetics of the reaction is consistent with a ping-pong bi-bi mechanism and the parameters of the respective kinetic equations are reported. Compared with native Cy3glc, the liposolubility, pH resistivity and thermostability of Cy3glc-C12 were significantly improved. The growth kinetics of *Bifidobacteria* and *Lactobacillus* was established based on the Logistic equation, and Cy3glc-C12 could promote their proliferation especially during the logarithmic growth, in which lower pH and more bacteria population were found compared with those of media without anthocyanins. This research provided a reference for the industrial production of Cy3glc-C12 and extended its application to natural products in lipophilic systems.

## 1. Introduction

Anthocyanins are a class of water-soluble natural colorants and widely distribute in fruits, vegetables, and flowers [1]. Up to now, there are more than 500 kinds of anthocyanins and 23 aglycons [2]. During the past decades, in vitro and in vivo studies have revealed that anthocyanins have health-promoting effects, including antioxidant, antineoplastic, anticarcinogenic, anti-inflammatory, antibacterial, and hepato-protective activities [3,4,5,6]. In our previous study, anthocyanins from black rice (recovery: 0.86%, purity: 95.93%) were extracted by buffer solutions, and were purified using membrane separation and resin adsorption [7]. The structures of anthocyanins from black rice were characterized as cyanidin-3,5-diglucoside, cyanidin-3-glucoside, cyanidin-3-rutinoside, peonidin-3-glucoside and peonidin-3-rutinoside by LC-MS/MS analysis [8]. Among them, cyanidin-3-glucoside is the most abundant monomer up to 85% [8,9,10]. Meanwhile, we confirmed the anthocyanins from black rice could promote the proliferation of *Bifidobacteria* and *Lactobacillus* and be metabolized into phenolic metabolites [10].

Now, anthocyanins have attracted increasing attention in the use as natural food colorants due to their attractive colors, toxicological safety and beneficial health effects [4]. However, anthocyanins are sensitive to pH, temperature, light, and oxidant and metal ions, which limit their application in the food industry [2,11,12]. Various methods, such as acylation [13], microencapsulation [14], and adding auxiliary compounds [15,16], have been attempted to increase the stability of anthocyanins in past decades. Therefore, chemical and enzymatic acylation have made substantial progress in improving the liposolubility and stability of anthocyanins in recent years [17,18,19,20,21,22,23]. Procyanidin B4 and malvidin-3-glucoside stearic acid derivatives were synthesized and characterized [17], and cyanidin-3-glucoside from blueberry was chemically acylated with lauric acid in vitro to produce Cy3glc-C12, in which the reaction (acylation rate: 30.78%) was continuously stirred for 48 h at 4 °C in DMF with Cy3glc (20 mg) and acyl donors, including lauric acid (1 equiv), 1-(3-dimethylaminopropy)-3-ethycarbodiimide (1.5 equiv) and N-hydroxybenzotriazole (1 equiv) [18]. Obviously, low acylated yield was obtained in the chemical acylation of Cy3glc with complex reaction conditions. Meanwhile, the acylated derivative was difficult to separate and purify from the reaction mixture.

In addition, enzymatic acylation of anthocyanins, with mild reaction conditions, high conversion yield, and recyclable materials, has been a better method [19]. In the previous studies, the transesterification reaction (conversion yield: 91%) between black rice anthocyanins and acyl donors (methyl benzoate, methyl salicylate, and methyl cinnamate) was stirred at 30 rpm under vacuum (900 mbar) in 5 mL pyridine for 48 h at 40 °C, using 1 g Novozym 435 lipase [20]. Although the conversion yield was high, pyridine, as a toxic solvent, could affect the safety of acylated products. To compensate for the solvent toxicity of pyridine, tert-butanol, tert-amyl alcohol, and acetonitrile with high hydrophobicity were used in direct esterification, such as malvidin-3-glucoside (yields: 22–40%) [21,22] and cyanidin-3-glucoside with different saturated fatty acids (yields: 25–47%) [23]. In the direct esterification, saturated fatty acids with different carbon chains were chosen as the acyl donors from liquid acid to solid acid. In this case, one hundred molar equivalents of acyl donors compared with anthocyanins were added to make the direct esterification go forward and compensate for the effect of fatty acids with different states on the conversion yield. However, excessive acyl donors (100 equiv.) brought a challenge to the subsequent separation and purification process.

Current research progresses on the enzymatic acylation of cyanidin-3-glucoside by direct esterification reaction mainly focus on its synthesis, identification, evaluation of stability, and in vitro antioxidant capacities [18,23]. There is little information about the effects of reaction conditions, such as the molar ratio, enzyme addition, reaction temperature, and time. In addition, the kinetics of this enzymatic synthesis is less reported while the proliferative effect of the acylated derivative on intestinal probiotics is rarely studied. The existing experimental results are of limited help to the practical application of industrialization, and the research on whether the product has the original bioactivity is still insufficient. In order to obtain an efficient, industrializable, environmentally friendly acylation route, the direct esterification reaction was chosen for enzymatic acylation of Cy3glc, in which tert-amyl alcohol with low toxicity and high hydrophobic coefficient was used as the reaction medium. A Box–Behnken design was applied to evaluate the effects of reaction factors, then Cy3glc-C12 was separated by column chromatograph and characterized by HPLC-MS/MS and NMR. The detailed kinetics of this reaction was studied by Michaelis–Menten model and various kinetic parameters were determined. Its physicochemical properties including liposolubility, UV–VIS absorbance property, and thermostability, were also studied. Furthermore, the potential proliferative effect of Cy3glc-C12 on *Bifidobacteria* and *Lactobacilli* were investigated by determining the bacterial populations, media pH, growth kinetics, and metabolic products in vitro.

## 2. Materials and Methods

### 2.1. Materials and Reagents

Black rice (*Oryza sativa* L.) from Yanshou County was purchased from Carrefour Supermarket (Hefei, China). Lauric acid, tert-amyl alcohol, and 4 Å molecular sieves (MS) were purchased from Aladdin (Shanghai, China). *Candida antarctica* lipase B (*Ca*lB, Novozym 435) (≥10,000 U·g^−1^, recombinant, expressed in *Aspergillus niger*) was purchased from Novozymes (Copenhagen, Denmark). *Bifidobacterium infantis* (CICC 6069), *Bifidobacterium adolescenties* (CICC6070)*, Bifidobacterium bifidum* (CICC 6071), and *Lactobacillus acidophilus* (CICC 6096) were purchased from China Center of Industrial Culture Collection (Beijing, China). The BS basal nutrient medium (pH 7.2) and MRS basal nutrient medium (pH 5.7) were purchased from Hope Bio-Technology Co., Ltd. (Qingdao, China).

### 2.2. Enzymatic Synthesis of Cy3glc-C12

Anthocyanins extract from black rice (purity 96%, in which Cy3glc of 85%) were obtained as the method described previously [7]. Anthocyanins and lauric acid of different molar ratios were added to tert-amyl alcohol to give a total volume of 10 mL, followed by MS 4 Å (100 g/L) and different amounts of *Ca*lB in a screw-capped glass reaction bottle. The mixture was shaken in a thermostatic oscillator (100 rpm) at different reaction temperatures and times. The reaction mixture was analyzed using a Waters E2695 high-performance liquid chromatograph HPLC system (Water Corp., Milford, MA, USA), equipped with a Waters 2998 diode array detector and an Ultimate XB-C18 reversed-phase column (5 μm, 250 × 4.6 mm). The detailed procedure is described in the Supplementary Materials, and the conversion yield is calculated by Equation (1):

(1) $$CY\;(\%)=\frac{A_{1}}{A_{0}{+A}_{1}}\times 100$$

where *CY* is the conversion yield, *A*_0_ is the Cy3glc peak area and *A*_1_ is the Cy3glc-C12 peak area.

### 2.3. Response Surface Methodology (RSM) Optimization Design

A three-level, four-variable Box–Behnken design (BBD) RSM model was employed. Based on the elementary single-factor experiments (Figure S1), the levels of four-variables were demonstrated in Table S1a. The optimization model of conversion yield is expressed in the following quadratic polynomial regression Equation (2):

(2) $$Y=\beta_{0}+\sum_{i}^{k}\beta_{i}X_{i}+\sum_{j}^{k}\beta_{ii}X_{i}^{2}+\sum_{i}\sum_{j}^{k}\beta_{ij}X_{i}X_{j}$$

where *Y* is the conversion yield; *X_i-j_* are molar ratio (*X*_1_), *Ca*lB addition (*X*_2_), reaction temperature (*X*_3_) and time (*X*_4_); *k* is the number of tested variables (*k* = 4); *β*_0_ is the constant term; *β_i_* is the linear coefficient; *β_ii_* is the quadratic coefficient and *β_ij_* is the interactive term coefficient.

### 2.4. Isolation of Cy3glc-C12 by Filtrate Extraction and Semi-Preparative HPLC

The isolation and purification of Cy3glc-C12 were carried out according to the previous reports [19]. After removal of *Ca*lB from the mixture, tert-amyl alcohol was then eliminated by a rotary vacuum evaporator at 60 °C. The residue was redissolved in acidified methanol solutions (with 2% HCl) and excess lauric acid was then extracted with heptane. The methanol fraction was, in turn, concentrated, diluted in 20% of aqueous acidified MeOH, and purified by a semi-preparative HPLC system loaded with an EP-C18M reversed-phase silica gel (10 μm, 250 × 10 mm) thermostated at 40 °C. The solvents, wavelengths, and mobile phase gradient were the same as described above in Supplementary Materials. The injection volume was 5 mL while the flow rate was 10 mL/min. The collection solution of Cy3glc-C12 was evaporated at 35 °C to remove organic solvents, freeze-dried, and stored at −80 °C for further use.

### 2.5. Structural Identification of Cyanidin-3-Glucoside Lauryl Ester

#### 2.5.1. Analysis by ESI-MS/MS

A SCIEX ExionLC™ system (SCIEX Corp., Shanghai, China) with a X500R QTOF mass detector was employed for analysis. The ESI ion source parameters were as follows: curtain gas, 30 Psi; ion spray voltage, 5500 V; temperature, 550 °C; ion source gas 1, 55 Psi; ion source gas 2, 55 Psi. The mass spectrometer was operated in a positive ion mode by scanning ions between 100 and 1000 m/z.

#### 2.5.2. Analysis by Nuclear Magnetic Resonance (NMR)

The freeze-dried Cy3glc and Cy3glc-C12 were analyzed with an Agilent VNMRS600 NMR spectrometer (CA, USA) operating at 600.08 and 150.56 MHz for ^1^H and ^13^C. The ^1^H and ^13^C spectra were recorded in CD_3_OD + TMS, and the spectra were processed with MestReNova 10.0 software (Mestrelab Research, Santiago de Compostela, Spain).

### 2.6. Kinetic Model and Parameters of Enzymatic Synthesis of Cy3glc-C12

Kinetics of enzymatic reactions can be described by the well-known Michaelis–Menten model (Equation (3)). For reactions having two substrates and two products (bi-bi reactions), its application is quite complicated since various mechanisms can be considered according to the order and rate of binding both substrates to the enzyme active sites and releasing the products from them:

(3) $$v=\frac{v_{\max}\left[A\right]\left[B\right]}{K_{\mathrm{ma}}\left[B\right]{+K}_{\mathrm{mb}}\left[A\right]+\left[A\right]\left[B\right]}$$

The equation can be expressed as Lineweaver–Burk double reciprocal equation:

(4) $$\frac{1}{v}=\frac{K_{\mathrm{ma}}}{v_{\max}}\frac{1}{\left[A\right]}+\frac{K_{\mathrm{mb}}}{v_{\max}}\frac{1}{\left[B\right]}+\frac{1}{v_{\max}}$$

where *v* is the initial reaction rate; *v*_max_ is the maximum reaction rate; *K*_ma_ is the kinetic constant of lauric acid; *K*_mb_ is the kinetic constant of Cy3gl; [A] is the lauric acid concentration; [B] is the Cy3glc concentration.

In this experiment, substrate concentration effects were analyzed by Ping-Pong Bi-Bi mechanism. In each group, 0.10, 0.15, and 0.20 mmol Cy3glc were added to 10 mL tert-amyl alcohol, followed by 1, 2, 3, 4 and 5 mmol lauric acid, respectively. The reactions were performed with 0.15 g *Ca*lB and 1 g MS 4Å of at 56 °C for 1 h. After the reaction finished, the conversion yield was determined by HPLC analysis, and the initial reaction rate is calculated by Equation (5):

(5) $$v=\frac{C_{0}-C_{t}}{m\times t}$$

where *v* is the initial reaction rate (mmol/g·min), *C*_0_ is the initial Cy3glc concentration (mmol/L), *C*_t_ is the Cy3glc concentration at time *t* (mmol/L), *m* is the *Ca*lB mass (g), and *t* is the time (min).

### 2.7. Properties of Cyanidin-3-Glucoside Lauryl Ester

#### 2.7.1. Lipophilic Property

The lipophilicity of Cy3glc from black rice and its acylated derivate Cy3glc-C12 were evaluated by the octanol-water partition coefficient (log P) following the methods described previously [24].

#### 2.7.2. UV–VIS Absorbance Property

In order to simulate a lipophilic system, Cy3glc and Cy3glc-C12 were dissolved in an aqueous 0.1 mol/L SDS solution (pH 3, 5, and 7) to reach a final concentration of 0.1 mmol/L. The detailed procedure is described in the Supplementary Materials.

#### 2.7.3. Thermostability

The effect of temperature on color stability of the anthocyanin solution was determined in a water bath at 65, 80, and 95 °C. After 2, 4, 6, 8, 10, and 12 h, the absorbance at the maximum wavelength (λ_max_) were measured with a 4802 UV–VIS double beam spectrophotometer (Unico Inc., Shanghai, China). The detailed procedure is described in the Supplementary Materials.

### 2.8. Effect of Cy3glc-C12 on the proliferation of *Bifidobacteria* and *Lactobacillus*

#### 2.8.1. Sample Preparation and Cultivation of the Strains

BS or MRS agar and broth were used as media for isolation and purification of *Bifidobacteria* and *Lactobacillus* by repeated plate streaking. Then, *B. infantis* CICC 6069, *B. adolescentis* CICC 6070, *B. bifidum* CICC 6071, and *L. acidophilus* CICC 6096 were cultivated in BS or MRS broth with an incubation level of 1% (about 1.5 × 10^6^ organisms). Purified Cy3glc and Cy3glc-C12 (density > 1 g/cm^3^) were sterilized with a filter and added to the media to reach an optimized concentration of 1.0 mg/mL based on our early report [10]. Aliquots of BS or MRS broth with equal amounts of inoculation without samples were prepared as matrix blank samples. For all runs, the culture media were incubated at 37 °C for 36 h under anaerobic conditions (85% N_2_, 10% CO_2_, and 5% H_2_) using an anaerobic chamber (Wanrui Laboratory Equipment Co., Model YQX-III, Shanghai, China).

#### 2.8.2. Calculation of Kinetic Parameters and OD_600_ Values for *Bifidobacteria* and *Lactobacillus* Growth

Bacterial growth during 36 h at 37 °C was monitored by measuring the optical density at 600 nm (OD_600_) using a spectrophotometer (Unico UV-4802, China). The *μ* value (specific growth rate) was calculated from the slope of the graph. Bacterial growth period, including the lag, log, and stationary phases, were studied by means of the Logistic equation:

(6) $$\frac{dX}{dt}{=\mu }_{m}X\left(1-\frac{X}{X_{m}}\right)$$

The equation could be integrated as:

(7) $$X=\frac{X_{0}X_{m}e^{\mu_{m}t}}{X_{m}-X_{0}{+X}_{0}e^{\mu_{m}t}}$$

where *X* is the bacterial OD_600_ at time *t*, *X*_0_ is the initial bacterial OD_600_, *X_m_* is the maximum bacterial OD_600_, *t* is the fermentation time (h), *μ_m_* is the maximum specific growth rate (h^−1^), and $\frac{dX}{dt}$ is the bacterial growth rate.

#### 2.8.3. Determination of Bacterial Metabolites and pH Values of Culture Media

The fermentation broth was sampled to measure the media pH at 0, 6, 12, 18, 24, 30, and 36 h, which reflected the acid production of four probiotics. Meanwhile, the corresponding bacterial metabolites were analyzed by GC-MS according to recent publication [10]. The detailed procedure is described in the Supplementary Materials.

### 2.9. Statistical Analysis

The data were analyzed using SPSS 20.0 (SPSS Inc., New York, NY, USA), and the least significant difference (LSD) post hoc test was conducted. Differences were considered statistically significant as *p* < 0.05. The results were presented as means ± standard deviation (n = 3).

## 3. Results and Discussion

### 3.1. Model Fitting of Lipase-Catalyzed Synthesis of Cy3glc-C12

The response of *Y*, representing Cy3glc acylation yield (%), was obtained under 29 different combinations in a BBD configuration, of varying levels of molar ratio, enzyme addition, reaction temperature, and time (Table S1b). The modeled Cy3glc acylation yield ranged from 48.19 to 67.32% in Table S1b. The *R*^2^ values, *F*-values, and *p*-values were calculated by analysis of variance in Table S1c. The *R*^2^ value of the model was 0.9885, suggesting that the second-order polynomial model fitted the experimental data well, with significance at a 95% confidence level. The response of the model was statistically acceptable due to significant regression (*p*_m_ < 0.05) and non-significant lack of fit (*p*_lf_ > 0.05). After fitting the observed data to multiple models, it showed that the relation between acylation yield (response) with independent variables were suitably described with a quadratic polynomial model as in Equation (8):

(8) $$Y=-348.52+4.73X_{1}+2.86X_{2}+6.82X_{3}+5.19X_{4}-0.005X_{1}X_{2}+0.013X_{1}X_{3}\;-0.001X_{1}X_{4}+0.030X_{2}X_{3}-0.034X_{2}X_{4}-0.050X_{3}X_{4}-0.048X_{1}^{2}\;-0.102X_{2}^{2}-0.059X_{3}^{2}-0.031X_{4}^{2}$$

### 3.2. Optimization of Reaction and Model Validation

**Figure 1:**
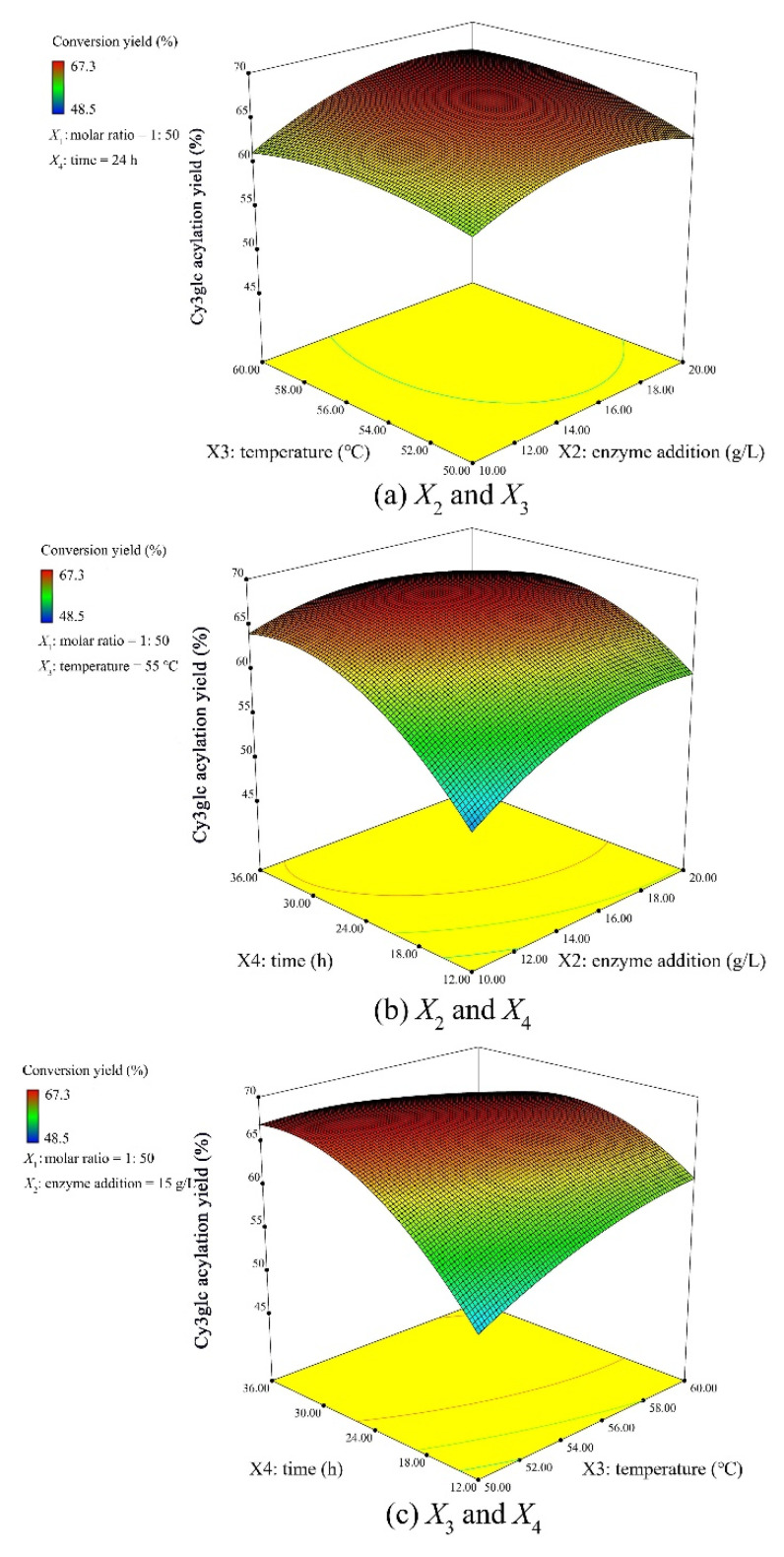
Response surface plots of the interactions between independent variables (**a**) enzyme addition *X*_2_ and temperature *X*_3_, (**b**) enzyme addition *X*_2_ and time *X*_4_, and (**c**) temperature *X*_3_ and time *X*_4_ on Cy3glc acylation yield (%).

Based on the experimental results, the Design Expert software (Stat-Ease Inc., Minneapolis, Minnesota, USA) predicted the optimal levels of the independent variables with desired maximum Cy3glc acylation yield (Figure 1). Therefore, a molar ratio of 1:56, enzyme addition of 16.5 g/L, temperature of 56 °C, and time of 28 h were chosen as the optimized independent variables (*Y* = 69.2%). To verify the validity and reproducibility of the model, experiments were performed under the predicted optimal conditions and the result was 68.7 ± 2.1%, suggesting the optimal conditions were credible.

In this study, the optimized enzymatic acylation of Cy3glc has a higher conversion yield than the previously reported acylation of anthocyanins. Malvidin-3-glucoside with different saturated fatty acid chain lengths using an enzymatic synthesis were obtained with low yields (22–40%) [21]. Zhao et al. described direct acylation of cyanidin-3-glucoside with lauric acid in blueberry, which was essentially chemical acylation with an acylated yield of 30.8% [18]. On the other hand, the optimal reaction conditions were more economical and efficient than the conditions mentioned in previous studies. The reaction temperature and molar ratio (acyl donor: substrate) was usually chosen as 60 °C and 100:1, such as malvidin-3-glucoside with stearoyl chloride [17], oleic acid [22], and saturated fatty acids of different chain lengths [21], delphindin-3-glucoside with palmitic acid [25], and cyanidin-3-galactoside with lauric acid [26]. Anthocyanins as reaction substrates could be easier to destroy at 60 °C over 24 h than the optimized reaction temperature of 56 °C. Excessive acyl donors might prevent anthocyanins from being fully dissolved in the reaction media, which resulted in the actual concentration of anthocyanins being lower than the apparent concentration, thus reducing the conversion yield [27]. In addition, the optimized enzymatic acylation was environmentally friendly, in which *Ca*lB, lauric acid, tert-amyl alcohol, and the 4 Å molecular sieve could be recycled [28]. Our work provided a reference for industrial production of Cy3glc-C12, which was mainly due to the enzymatic acylation method. The enzymatic method could be applied in industry because of the high yield and recoverable feature. The maximum conversion yield (68.74 ± 2.11%) was obtained with a molar ratio (substrate: donor) of 1:56, 16.5 g/L of Novozym 435, temperature of 56 °C, and time of 28 h via the acylation occurring at the 6”-OH position of the glucoside. The detailed amount of Cy3glc-C12 depends on the amount of Cy3glc under the above conditions. In this work, 0.05 mmol Cy3glc was added while 0.034 mmol Cy3glc-C12 was produced. The conversion yield might fluctuate in the industrial production of Cy3glc-C12. Overall, the purpose of this optimization was to obtain an enzymatic acylation route for industrial applications.

### 3.3. Identification of Cy3glc-C12 by ESI-MS/MS and NMR

**Figure 2:**
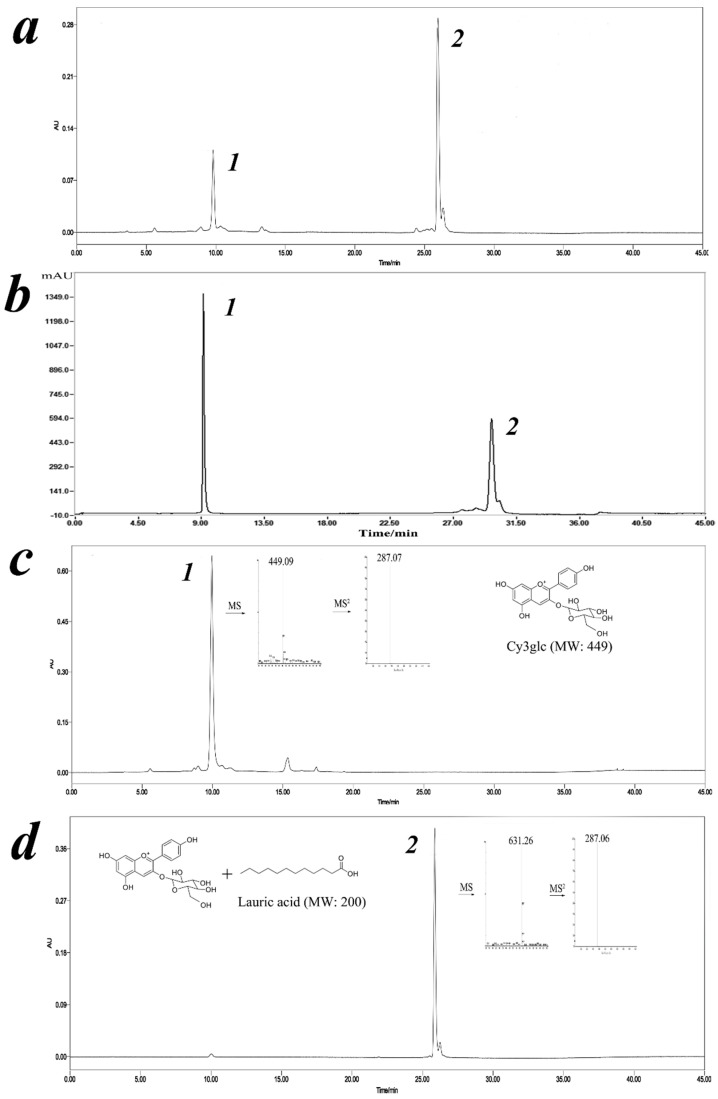
HPLC chromatogram of the reaction mixture to determine the Cy3glc acylation yield (**a**), semi-preparative liquid chromatogram of the reaction mixture to obtain Cy3glc and Cy3glc-C12 (**b**), and HPLC-MS/MS spectra of Cy3glc (**c**) and Cy3glc-C12 (**d**).

The mixture was analyzed by LC-DAD-ESI/MS in positive ion mode and its characterization was performed bearing their molecular ions and related fragments (Figure 2). A new peak around 26 min was identified as corresponding to a Cy3glc molecule attached to a lauric acid moiety according to its full MS spectrum, [M]^+^ at m/z 631. In addition, the Cy3glc–lauric acid conjugate revealed an MS^2^ fragment, [M-344] ^+^ at m/z 287, which was in agreement with the Cy3glc aglycone structure (loss of the glucose residue attached to the lauric acid group). This means that acylation of Cy3glc occurred preferentially in the OH groups of the glucose moiety instead of any hydroxyl groups from the flavylium core.

The product was further characterized by ^1^H and ^13^C NMR in CD3OD+TMS (Table 1). The enzymatic reaction yielded only a monoester and its regioselectivity was constant, showing that acylation always took place on the primary hydroxyl group present on the glycoside moiety of the molecule (C6ʹʹ-OH). The location of the acylated site has been well documented in previous studies for a range of acyl donors [24,26]. This was confirmed by comparing downfield and upfield shifts between Cy3glc and the Cy3glc-C12 in the same solvent. ^13^C NMR spectra showed that the acylation on the 6ʹʹ-OH position led to an upfield of the C-6ʹʹ signal by approximately 2.7 ppm (∼62.21 to ∼64.90 ppm) due to the resonance effect towards the carbonyl of the newly generated ester.

### 3.4. Kinetics of Enzymatic Synthesis of Cy3glc Lauryl Ester

**Figure 3:**
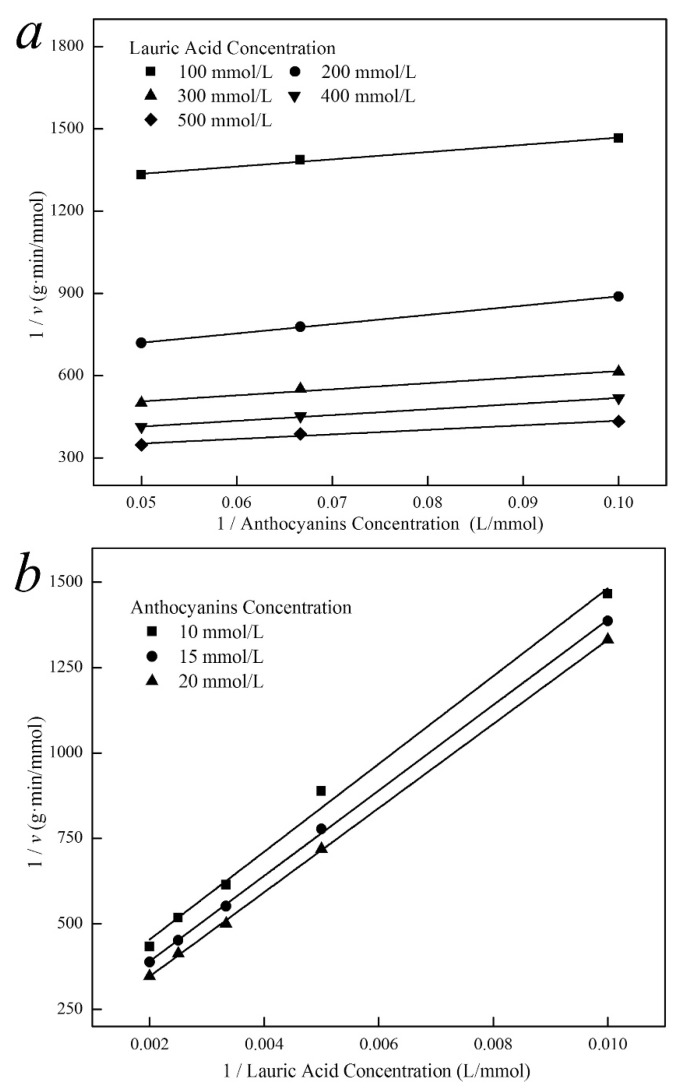
Double-reciprocal fit plot of the initial reaction rate with Cy3glc concentration (**a**) and lauric acid concentration (**b**) (n = 3).

To study the effect of substrate concentration, the reaction kinetics was determined under the optimized conditions: *Ca*lB 16.5 g/L, reaction temperature 58 °C and time 28 h. As shown in Figure 3, no substrate inhibition was observed as Cy3glc concentration was less than 25 mmol/L. According to the requirements of dynamic research, the following assumptions were made [12]: Firstly, *Ca*lB concentration was very low compared with substrate concentration, so that the formation of substrate complex would not cause significant changes in substrate concentration. Secondly, the production of Cy3glc-C12 and water was irreversible at the beginning of the reaction and when the initial reaction velocity was measured. Thirdly, the concentration of lipase Novozym 435 remained stable during the reaction. Therefore, the reaction process was consistent with the double substrate Ping-Pong mechanism.

A series of basically parallel lines were obtained when the reciprocal of the 1/*v* was used as the ordinate and the reciprocal of Cy3glc or lauric acid was used as the abscissa for the double-reciprocal fitting curve (Figure 3). Thus, the kinetic equation of acylation reaction between Cy3glc and lauric acid catalyzed by lipase could be calculated with Equation (9):

(9) $$v=\frac{0.0226[A][B]}{2838.56[B]+54.22[A]+[A][B]}$$

The catalyst *Ca*lB (E) was firstly combined with lauric acid (A) to produce a binary complex EA. A new complex EAcBP (Ac: lauric acid residue, P: water) was then formed by the frequent collision between EA and Cy3glc (B). The intermediate EAcB appeared after the release of P, and EQ (Q: Cy3glc-C12) was successfully synthesized after the acylation. Finally, the acylated product Q was separated from the binary complex EQ, and the enzyme E was recycled. The enzymatic acylation of Cy3glc with lauric acid to produce Cy3glc-C12 is a double substrate and double product reaction, which is operated by a Ping-Pong mechanism.

### 3.5. Properties of Cyanidin-3-Glucoside Lauryl Ester

#### 3.5.1. Lipophilic Property

**Figure 4:**
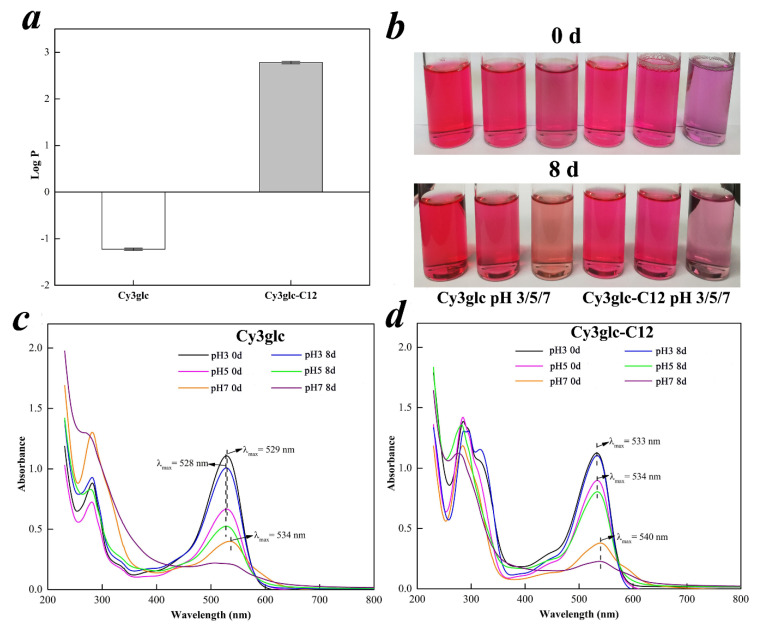
Lipophilic property (**a**), color feature (**b**), and UV-VIS absorbance property of Cy3glc and Cy3glc-C12 (**c** and **d**) during storage for eight days in the dark at pH 3, 5, and 7.

In this experiment, the lipophilicity of Cy3glc and Cy3glc-C12 was evaluated by octanol/water partition coefficient (log P) (Figure 4a). The log P significantly increased from –1.23 to 2.78 after the reaction, indicating that lipophilic Cy3glc-C12 was transformed from hydrophilic Cy3glc. The result was corresponding with the peak delay of Cy3glc-C12 (Figure 2a), in which the retention time mainly depended on substance polarity in HPLC analysis with reversed phase C18 column [29]. Overall, the enzymatic acylation could be a potential method to improve the lipophilicity of anthocyanins.

#### 3.5.2. UV-VIS Absorbance Property

In order to mimic a lipophilic medium, the aqueous SDS model solutions were used to dissolve the anthocyanins. Their chromatic features were photographed (Figure 4b), and all optical wavelengths at room temperature over a period of eight days were preliminarily determined by UV–VIS (Figure 4c,d). In Figure 4b, the color of Cy3glc-C12 was slightly purple on the basis of redness in visual appearance, and significantly different from the color of the native Cy3glc. Their color faded with increasing time, especially in the pH 7 SDS model solutions. Clearly, the absorption showed strong bathochromic shift effect at different pH after the enzymatic acylation (Figure 4c,d). *λ*_max_ increased from 529 nm (Cy3glc) to 533 nm (Cy3glc-C12) at pH 3 while *λ*_max_ increased from 528 nm to 534 nm at pH 5. At pH 7, compared with the initial curve, Cy3glc lost its characteristic absorption peak after 8 days. However, Cy3glc-C12 still kept its feature after the same equilibrium period. Overall, cy3glc-C12 had better color intensity and stronger resistance to pH change in the presence of acyl groups linked to the sugar moieties^4^.

#### 3.5.3. Thermostability

With rising temperature, an increase in rate constant *k*, and a corresponding decline in half-life *t*_1/2_ were recognized in Table 2. The rate constant *k* of Cy3glc-C12 were 0.074 (pH 3), 0.106 (pH 5) and 0.243 h^−1^(pH 7) at 95 °C in a water bath, in which the corresponding half-life *t*_1/2_ was 9.44, 6.60, and 2.86 h, respectively. Compared with Cy3glc, a lower *k* and higher *t*_1/2_ value of Cy3glc-C12 indicated that its thermostability was significantly increased by enzymatic acylation. Furthermore, the similar trend in kinetic parameters was obtained at the other temperature (65 and 80 °C). Typically, a sandwich structure was formed in the presence of poly-acylation, protecting the molecule from water nucleophilic attack by the package of acyl groups [13]. The intramolecular colorization of anthocyanins could also be achieved, reducing the formation of chalcone or methylamine structure [18]. Altogether, enzymatic acylation of anthocyanins could be applied as an efficient method to significantly increase their thermal stability.

### 3.6. Proliferative Effect of Cy3glc-C12 on *Bifidobacteria* and *Lactobacillus*

**Figure 5:**
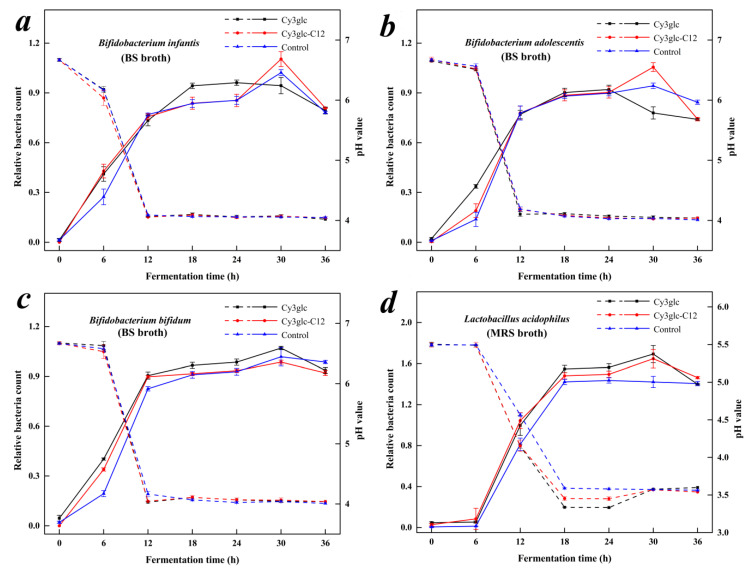
The change of strains growth (solid line) and pH value (dotted line) during the fermentation with Cy3glc or Cy3glc-C12. BS, and MRS broth without anthocyanins were kept as the control groups (CG). Error bars represent the standard deviations of the mean of three replicates (n = 3). (**a**) *B. infantis* growth in BS broth; (**b**) *B. adolescentis* growth in BS broth; (**c**) *B. bifidum* growth in BS broth; (**d**), *L. acidophilus* growth in MRS broth.

In this experiment, OD_600_ value with the corresponding pH was adopted to monitor the growth of *Bifidobacteria* and *Lactobacillus* (Figure 5), and their growth kinetic parameters were calculated (Table 3). In Figure 5a, *B. infantis* showed similar growth curves in the media with Cy3glc-C12 and the CG, in which its relative bacteria count (RBC) increased from 0 to 30 h and decreased after incubation for 30 h. Meanwhile, *B. infantis* had a higher *X*_m_ (0.960 ± 0.056) and lower *μ*_m_ (0.319 ± 0.079 h^−1^) in the media with Cy3glc than those of the CG (*p* < 0.05). The results indicated that Cy3glc enabled *B. infantis* to rapidly enter the log phase from the lag phase, so that *B. infantis* could effectively utilize the media resource in the limited time, which also explained its advanced decline phase and faster decay rate under the circumstance of limited media resources. *B. infantis* is a common probiotic strain that is associated with a healthy gut and digestion, and it mainly exists in the gut flora of infantis [30]. Lipophilic Cy3glc-C12 was difficult to dissolve in BS broth so that it was not well contacted and metabolized by *B. infantis* at first, resulting in different proliferative effect between Cy3glc and Cy3glc-C12. With the increase of *B. infantis*, Cy3glc-C12 could be utilized and further promoted the proliferation of *B. infantis* according to a maximum RBC after incubation for 30 h.

In the growth of *B. adolescentis*, there was a significant decrease in *μ*_m_ between the media with Cy3glc (0.390 ± 0.038 h^−1^) or Cy3glc-C12 (0.536 ± 0.018 h^−1^) and the CG (Table 3). Even though the media with Cy3glc had the lowest *μ*_m_ value, to some extent, Cy3glc-C12 also enabled *B. adolescentis* to enter the log phase from the lag phase (Figure 5b). Compared with *B. infantis*, *B. adolescentis* had a better capacity in utilizing Cy3glc or Cy3glc-C12, which was reflected in the advanced decline phase after incubation for 24 h (Cy3glc) and a significant *μ*_m_ value (Cy3glc-C12). Even so, the proliferative effect of Cy3glc-C12 on *B. adolescentis* was mainly found in the later stage of its logarithmic growth based on the maximum RBC after incubation for 30 h, which was similar with that of *B. infantis*.

*B. bifidum* showed a higher RBC value in the media with Cy3glc or Cy3glc-C12 than that of the CG during the incubation from 0 to 12 h (Figure 5c). The proliferative effect of Cy3glc on *B. bifidum* was similar with those of *B. infantis* and *B. adolescentis*, in which *B. bifidum* could rapidly enter the log phase from the lag phase and utilize the limited resources to grow, resulting in a significant increase in *X*_m_ (0.979 ± 0.007) and decrease in *μ*_m_ (0.477 ± 0.025 h^−1^). The growth curve of *B. bifidum* in the Cy3glc-C12 media was similar to that of Cy3glc media, indicating that both of them played a similar role in promoting the proliferation of *B. bifidum*. However, the *μ*_m_ value in the Cy3glc-C12 media (0.671 ± 0.044 h^−1^) was significantly higher than that of the CG (0.566 ± 0.033), revealing that the effect of Cy3glc-C12 focused on the logarithmic growth of *B. bifidum*, which differed from those of *B. infantis* and *B. adolescentis*. *B. bifidum* is mainly found in the intestinal flora of adults, and its uptake capacity of lipophilic Cy3glc-C12 is better than the other *Bifidobacteria*, leading to the advanced time of proliferative effects.

As for *L. acidophilus*, its log phase appeared after incubation for 6 h and lasted from 6 to 18 h, which significantly differed from those of *Bifidobacteria* (Figure 5d). The growth curves of *L. acidophilus* were similar in the media with Cy3glc and Cy3glc-C12, and they had higher RBC values in the log phase compared with the CG. In addition, the decline phase of *L. acidophilus* was observed in the media with samples after incubation for 30 h while *L. acidophilus* was still in the stationary phase in the CG. Higher *X*_m_ values and lower *μ*_m_ values were simultaneously detected in the Cy3glc and Cy3glc-C12 media, indicating that Cy3glc and Cy3glc-C12 had same proliferative effects on *L. acidophilus*. *L. acidophilus* is not only found in the stomach, but also a major probiotic in the small intestine. Meanwhile, *L. acidophilus* could easily contact and metabolize lipophilic Cy3glc-C12 in an aqueous system in vitro, and its utilization capacity of Cy3glc-C12 was better than the three *Bifidobacteria* species.

Due to the liposolubility of Cy3glc-C12, its proliferative effect on *Bifidobacteria* and Lactobacillus was different from that of Cy3glc. Lipophilic Cy3glc-C12 was difficult to dissolve in BS or MRS broth and deposited at the bottom of the test tube. To begin with, three *Bifidobacteria* species could not contact Cy3glc-C12 as easily as Cy3glc. Therefore, Cy3glc could promote the proliferation of *Bifidobacteria* from making it rapidly enter the log phase from the lag phase and having a longer logarithmic growth after incubation for 24 h. As for Cy3glc-C12, with the growth of *Bifidobacteria*, Cy3glc-C12 could be gradually contacted and utilized in the log phase so that its proliferative effect was obviously observed in the middle and later log phase. On the other hand, due to the different species of intestinal probiotics, their capacity in utilizing lipophilic Cy3glc-C12 further influenced the growth curve, in which *L. acidophilus* > *B. bifidum* > *B. infantis* > *B. adolescentis*. Thus, there was no significant difference between the effect of Cy3glc and Cy3glc-C12 on the lag phase of *L. acidophilus*, and their contact and metabolization by *L. acidophilus* were nearly synchronous.

Their proliferative effects on *Bifidobacteria* and *Lactobacillus* could be explained from two aspects. Firstly, Cy3glc and Cy3glc-C12 could be broken down by *Bifidobacteria* and *Lactobacillus* into phenolic metabolites, such as benzoic acid, phenyllactic acid, and phenylalanine (Table 4), which could be used as sources to offer intestinal probiotics better growth conditions [10,31]. Secondly, pH in the media with Cy3glc and Cy3glc-C12 dropped faster than the control. Cy3glc-C12 metabolites, produced by *Bifidobacteria* and *Lactobacilli* after incubation for 24 h, were analyzed by GC-MS. About 14 organic acids produced by spontaneous degradation of cyanidin were detected in GC-MS. The results indicated that the type and content of Cy3glc-C12 metabolites were similar to that of Cy3glc metabolites in our previous report [10]. Understandably, Cy3glc and Cy3glc-C12 were metabolized into organic acids by the tested bacteria and the media pH inevitably dropped in the presence of organic acids. As *L. acidophilus*, the relative contents of main organic acids were benzoic acid (1.47), phenyllactic acid (6.67), and phenylalanine (4.50), respectively. Due to sugar-based connection, Cy3glc or Cy3glc-C12 were not readily absorbed by the digestive tract, but hydrolyzed to aglycones by the intestinal bacterial glycosidase or further degraded into phenolic acids [31,32,33]. An acidic environment (pH 4.5–6.5) is beneficial to the growth of *Bifidobacteria* and *Lactobacillus* [34]. Cy3glc and Cy3glc-C12 metabolites not only provided appropriate pH for the growth of intestinal probiotics, but also inhibited the growth of spoilage organisms [5].

*Bifidobacteria* decreased faster at their decline phase in Cy3glc and Cy3glc-C12 media, which could be attributed to two aspects: Firstly, the nutrients were quickly used up by *Bifidobacteria* due to the bioactivity of Cy3glc and Cy3glc-C12. After incubation for 24 h, essential nutrients were deficient in the medium, which might be responsible for their faster decay. Secondly, phenolic hydroxyl groups of Cy3glc and Cy3glc-C12 could react with mycoproteins or enzymes through hydrogen bonding, resulting in cytoplasm pyknosis and disintegration [5]. However, no significant difference of relative bacteria count was observed in Cy3glc and Cy3glc-C12 (*p* > 0.05) after 36 h of incubation. As for the hysteretic decline phase of *B. infantis* and *B. adolescentis* in Cy3glc-C12 media, it could be explained from the steric hindrance (the ability of probiotics to ingest Cy3glc-C12) and lipid solubility. The long carbon chain of Cy3glc-C12 could increase the steric hindrance, which limited the proliferative effect of Cy3glc-C12. On the other hand, the lipid solubility of Cy3glc-C12 limited its pathway from water-soluble media to intestinal probiotics. However, Cy3glc-C12 was easier to go through cell membranes of intestinal probiotics because of its lipid solubility (Figure 4a) [26].

Overall, Cy3glc-C12 displayed a similar influence to Cy3glc on proliferation of intestinal probiotics, further confirming well bioactive of Cy3glc-C12. Overall, the results revealed that Cy3glc-C12 could promote the growth of intestinal probiotics, and sustain as well proliferative effect as Cy3glc after the attachment of lauric acid to the 6″-OH position of the glucoside unit.

## 4. Conclusions

The optimal enzymatic acylation route of 68.7 ± 2.1% conversion yield was obtained by a Box–Behnken RSM design with anthocyanins: lauric acid (molar ratio) 1:56, *Ca*lB 16.5 g/L and molecular sieves 4 Å, 100 g/L in tert-amyl alcohol at 56 °C for 28 h. Cyanidin-3-(6′′-dodecanoyl)-glucoside was successfully produced via the acylation occurred at 6′′-OH position of the glucoside. The enzymatic acylation of Cy3glc with lauric acid to produce Cy3glc-C12 is a double substrate and double product reaction, which is operated by a Ping-Pong Bi-Bi mechanism. The liposolubility, pH resistance, and thermostability of Cy3glc-C12 were significantly improved after enzymatic acylation. Furthermore, Cy3glc-C12 could promote the proliferation of *Bifidobacteria* and *Lactobacillus* in the middle and later log phase and further be metabolized into phenolic acids. This research established a kinetic model for the enzymatic synthesis of lipophilic, thermostable, and anthocyanin-based pigments and revealed the proliferative effect of Cy3glc-C12 on intestinal probiotics.

## Figures and Tables

**Table 1:**
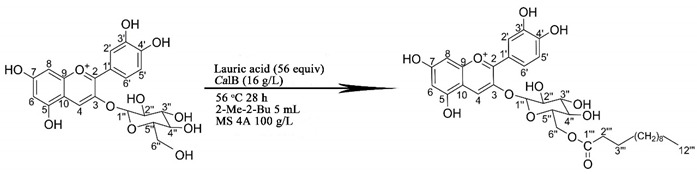
^1^H and ^13^C NMR data of Cy3glc and Cy3glc-C12 in CD_3_OD+TMS.

Position	Cy3glc		Cy3glc-C12	
	δ_H_ (ppm)*	δ_C_ (ppm)	δ_H_ (ppm)*	δ_C_ (ppm)
*Cyanidin aglycone*				
4	7.50 (s, 1H)	132.52	7.48 (s, 1H)	134.31
6	6.52 (s, 1H)	102.96	6.48 (s, 1H)	103.02
8	6.68 (s, 1H)	96.37	6.68 (s, 1H)	96.42
2′	6.94 (d, J = 1.9 Hz, 1H)	116.57	6.94 (d, J = 2.1 Hz, 1H)	116.40
5′	6.83 (d, J = 7.4 Hz, 1H)	117.18	6.85 (d, J = 7.5 Hz, 1H)	117.27
6′	7.35 (dd, J = 7.4, 2.1 Hz, 1H)	126.84	7.35 (dd, J = 7.4, 2.1 Hz, 1H)	125.99
*3-O-β-D-glucoside*				
1′′	5.07 (s, 1H)	102.50	5.02 (s, 1H)	101.16
2′′	3.66 (d, J = 5.1 Hz, 1H)	73.39	3.61 (d, J = 4.9 Hz, 1H)	73.47
3′′	3.44 (d, J = 4.9 Hz, 1H)	76.81	3.78 (d, J = 5.1 Hz, 1H)	76.90
4′′	3.40 (d, J = 5.1 Hz, 1H)	71.24	3.55 (d, J = 5.1 Hz, 1H)	70.82
5′′	3.45 (s, 1H)	78.04	3.72 (s, 1H)	75.60
6′′	3.82–3.67 (m, 2H)	62.21	4.31 (q, J = 12.6 Hz, 2H)	64.90
*6′′-O-dodecanoate*				
CH_2_ (2′′′)	/	/	2.33–2.26 (m, 2H)	34.45
CH_2_ (3′′′)	/	/	1.50 (s, 2H)	25.48
CH_2_ (C_4_-C_11_)	/	/	1.24–1.21 (m, 16H)	22.90–31.98
CH_3_ (C_12_)	/	/	0.88 (s, 3H)	14.22

**Table 2:** Kinetic parameters determined from the thermal-degradation assays of Cy3glc and Cy3glc-C12 in an aqueous SDS solution.

Indicators		Cy3glc			Cy3glc-C12		
		65 °C	80 °C	95 °C	65 °C	80 °C	95 °C
**pH3**	*k* (h^−1^)	0.078 ± 0.010	0.210 ± 0.004	0.347 ± 0.004	0.054 ± 0.010	0.066 ± 0.005	0.074 ± 0.005
	*t*_1/2_ (h)	9.04 ± 1.25	3.30 ± 0.06	2.00 ± 0.02	13.15 ± 2.19	10.48 ± 0.75	9.44 ± 0.61
	*E_a_* (kJ·mol^−1^)	52.11 ± 5.00 a			13.97 ± 3.23 e		
pH5	*k* (h^−1^)	0.150 ± 0.011	0.210 ± 0.011	0.344 ± 0.006	0.077 ± 0.005	0.096 ± 0.010	0.106 ± 0.011
	*t*_1/2_ (h)	4.65 ± 0.33	3.31 ± 0.18	2.01 ± 0.04	8.97 ± 0.60	7.29 ± 0.80	6.60 ± 0.74
	*E_a_* (kJ·mol^−1^)	28.70 ± 2.42 c			10.70 ± 1.53 e		
pH7	*k* (h^−1^)	0.179 ± 0.010	0.292 ± 0.004	0.342 ± 0.002	0.088 ± 0.004	0.203 ± 0.004	0.243 ± 0.011
	*t*_1/2_ (h)	3.88 ± 0.21	2.37 ± 0.03	2.03 ± 0.01	7.91 ± 0.33	3.41 ± 0.06	2.86 ± 0.14
	*E_a_* (kJ·mol^−1^)	22.57 ± 1.79 d			35.44 ± 1.56 b		

Data were obtained from three independent experiments and represented as the mean values ± SD (n = 3). Different lowercase letters indicate significant differences (*p* < 0.05) in the activation energy.

**Table 3:** Growth kinetic parameters of three *Bifidobacteria* and one *Lactobacillus* species.

Parameters	*X*_0_ (OD_600nm_)	*X*_m_ (OD_600nm_)	*μ*_m_ (h^−1^)	*R*^2^ of the Model
*Bifidobacterium infantis*				
CG	0.019 ± 0.003	0.847 ± 0.006	0.504 ± 0.023	0.999
Cy3glc	0.029 ± 0.012^ns^	0.960 ± 0.056^*^	0.319 ± 0.079^*^	0.974
Cy3glc-C12	0.015 ± 0.014^ns^	0.832 ± 0.029^ns^	0.501 ± 0.151^ns^	0.986
*Bifidobacterium adolescentis*				
CG	0.008 ± 0.002	0.889 ± 0.006	0.606 ± 0.019	0.999
Cy3glc	0.006 ± 0.002^ns^	0.915 ± 0.018^ns^	0.390 ± 0.038^*^	0.997
Cy3glc-C12	0.010 ± 0.001^ns^	0.901 ± 0.006^ns^	0.536 ± 0.018^*^	0.999
*Bifidobacterium bifidum*				
CG	0.008 ± 0.002	0.926 ± 0.010	0.566 ± 0.033	0.993
Cy3glc	0.018 ± 0.006^ns^	0.979 ± 0.007^*^	0.477 ± 0.025^*^	0.999
Cy3glc-C12	0.009 ± 0.010^ns^	0.927 ± 0.007^ns^	0.671 ± 0.044^*^	0.999
*Lactobacillus acidophilus*				
CG	0.001 ± 0.001	1.433 ± 0.006	0.805 ± 0.072	0.999
Cy3glc	0.001 ± 0.001^ns^	1.562 ± 0.025^*^	0.655 ± 0.054^*^	0.999
Cy3glc-C12	0.002 ± 0.001^ns^	1.495 ± 0.015^*^	0.609 ± 0.043^*^	0.999

Results are expressed as mean ± SD (n = 3). * Significantly different from the CG, *p* < 0.05 and ^ns^ not significantly different from the CG, *p* > 0.05.

**Table 4:** Relative content of Cy3glc-C12 metabolites produced by *Bifidobacteria* and *Lactobacilli* at 24 h of incubation under anaerobic conditions at 37 °C.

Compound (Relative Content)	Retention Time (min)	Molecular ion (m/z)	Intestinal Probiotics			
			*B. Infantis*	*B. Adolescentis*	*B. Bifidum*	*L. Acidophilus*
benzoic acid	11.26	179	1.05	1.18	1.14	1.47
Phenylacetic acid	12.39	193	0.03	0.01	0.02	0.01
Phenylpropanoic acid	15.00	104	0.10	0.31	0.29	0.14
Mandelic acid	16.18	179	0.02	0.05	0.05	0.05
4-Hydroxybenzaldehyde	16.59	223	0.07	0.06	0.05	0.16
Phenethylamine	17.94	174	0.02	0.03	0.13	0.02
4-Hydroxyphenylethanol	18.07	179	0.06	0.16	0.18	0.16
Phenyllactic acid	18.26	193	9.63	12.04	14.11	16.67
Phenylalanine	18.96	218	4.31	2.20	3.11	4.50
4-Hydroxybenzoic acid	19.01	267	0.06	0.05	0.07	0.16
4-Hydroxyphenylacetic acid	19.18	179	0.09	0.05	0.05	0.05
4-Hydroxyphenylpropionic acid	20.89	179	0.11	0.17	0.18	0.15
3-Methoxy-4-hydroxybenzoicacid	20.93	297	0.08	0.03	0.03	0.12
4-Aminobenzoic acid	21.79	266	0.02	0.03	0.03	0.01

## Supplementary Materials

The following are available online at https://www.mdpi.com/2079-7737/9/8/205/s1, Figure S1: Effect of reaction parameters on acylation yield of Cy3glc. molar ratio (**a**), enzyme addition (**b**), reaction temperature (**c**), and time (**d**), Figure S2: 1H, 13C NMR and 2D NMR spectrum (HSQC) for the synthesized compound Cy3glc-C12. (a) 1H NMR, (b) 13C NMR and (c) HSQC 2D NMR, Table S1: Experimental design, results, and variance analysis of response surface optimization. The supplementary materials included the methods about determination of cyanidin-3-glucoside acylation yield, measurement of lipophilicity, UV–VIS absorbance property, thermal stability assay and analysis of secondary metabolites by GC-MS. In addition, the results of single factor test, response surface test and NMR spectrum were also presented. Reference [9] is cited in the supplementary materials.
