# Supplementary material for: Kinetics of Enzymatic Synthesis of Cyanidin-3-Glucoside Lauryl Ester and Its Physicochemical Property and Proliferative Effect on Intestinal Probiotics
Source: Biology (Basel). 2020 Aug 4;9(8):205. doi: 10.3390/biology9080205 (PMC7465376; doi:10.3390/biology9080205)
Supplement: Supplementary file 1 [file biology-09-00205-s001.zip › biology-873177-supplementary.pdf]

Supplementary Materials

The supplementary materials included the methods about determination of cyanidin-3-glucoside acylation yield, measurement of lipophilicity, UV–VIS absorbance property, thermal stability assay and analysis of secondary metabolites by GC-MS. In addition, the results of single factor test, response surface test and NMR spectrum were also presented.

Determination of Cyanidin-3-Glucoside Acylation Yield

The reaction mixture was analyzed using a Waters E2695 high-performance liquid chromatograph HPLC system (Water Corp., Milford, MA, USA), equipped with a Waters 2998 diode array detector and an Ultimate XB-C18 reversed-phase column (5 µm, 250 × 4.6 mm). The analysis was carried out by a gradient elution of formic acid/water (5:95, *v*/*v*) as solvent A and acetonitrile as solvent B at a mobile phase flow rate of 1 mL/min. The gradient program of the mobile phase was: 0–5 min, 5–10% B; 5–10 min, 10% B; 10–17 min, 10–24% B; 17–27 min, 24–90% B; 27–30 min, 90% B; 30–35 min, 90–5% B; 35–45 min, 5% B. The injection volume was 10 µL and the column temperature was maintained at 40 °C. The peaks were monitored at 280 nm and 520 nm for all phenolic compounds and Cy3glc-LA. The acylation yield (*Y*) of Cy3glc was calculated by Equation (S1).

$$Cy3glc\ acylation\ yield\ (\%) = \frac{A_1}{A_0 + A_1} \times 100 \quad \text{(S1)}$$

where A_0 is the peak area of cyanidin-3-glucoside and A_1 is the peak area of cyanidin-3-glucoside–lauric acid conjugate.

Measurement of Lipophilicity

Briefly, acidified water (2%, *v*/*v* HCl) and n-octanol were mutually saturated with each other (1:3, *v*/*v*) for 24 h. The samples (2 mg) were dissolved in n-octanol saturated with acidified water (2 mL), and the absorbance (A_0) at 520 nm was determined. Subsequently, 2 mL of acidified water saturated with n-octanol was added, and the mixtures were vigorously shaken for 1 h, followed by centrifugation at 2500× *g* for 30 min. The absorbance (A_1) of n-octanol in the upper layer was measured at 520 nm. The octanol/water partition coefficient was calculated with Equation (S2):

$$\log P = \log A_1/(A_0 - A_1) \quad \text{(S2)}$$

UV–VIS Absorbance Property

In order to guarantee total solubilization, the stability of cy3glc and cy3glc-C12 were evaluated at the final concentrations of 0.1 mM dissolved in an aqueous 0.1 M SDS solution (pH 3, 5, and 7). The effect of temperature on color stability of the anthocyanin solution was determined in a water bath at 65, 80 and 95 °C. After 2, 4, 6, 8, 10, and 12 h, the absorbance at the maximum wavelength (λ_{max}) were measured with a 4802 UV–VIS double beam spectrophotometer (Unico Inc. Shanghai, China). The logarithms of ln (A/A_0) were plotted as function of time of heat treatment (t), where A_0 is the initial absorbance at λ_{max}, and A is the absorbance after the heating time at a selected temperature. The degradation processes followed pseudo-first-order reaction kinetics [35]. The rate constant (k) and half-life ($t_{1/2}$) were calculated by Equations (S3) and (S4):

$$\ln\left(\frac{A}{A_0}\right) = -kt \quad \text{(S3)}$$

$$t_{1/2} = \frac{\ln 2}{k} \quad \text{(S4)}$$

The activation energy (E_a) was estimated using the Arrhenius equation (Equation (S5)):

$$\ln k = \ln k_0 - \frac{E_a}{RT} \quad \text{(S5)}$$

where R is the universal gas constant (8.314 J·mol^{-1}·K^{-1}), k_0 is the frequency factor, and T is the absolute temperature (K).

The effect of pH on color stability was investigated during storage at room temperature for eight days. After 2, 4, 6, and 8 d, the absorbance-decay evolution at λ_{max} of cy3glc and its derivative in aqueous 0.1 M SDS solution at different pH were recorded by an UV–VIS spectrophotometer.

Thermal Stability Assay

Cy3glc and Cy3glc-LA were dissolved in an aqueous 0.1 mol/L SDS solution (pH 3, 5, and 7) to reach a final concentration of 0.1 mmol/L. The effect of temperature on their color stability was evaluated in a water bath at 65, 80, and 95 °C, respectively. The thermal degradation processes followed pseudo-first-order reaction kinetics. The rate constant k, half-life $t_{1/2}$ and activation energy E_a were calculated by Equations (S6)–(S8).

$$\ln\left(\frac{A}{A_0}\right) = -kt \quad \text{(S6)}$$

$$t_{1/2} = \frac{\ln 2}{k} \quad \text{(S7)}$$

$$\ln k = \ln k_0 - \frac{E_a}{RT} \quad \text{(S8)}$$

where A_0 is the initial absorbance, and A is the absorbance after the heating time at a selected temperature, t is the time of heat treatment (h), k_0 is the frequency factor (h^{-1}), R is the universal gas constant (8.314 J·mol^{-1}·K^{-1}), and T is the absolute temperature (K).

Analysis of Secondary Metabolites by GC-MS

The broth samples were respectively collected at 24 h of incubation. After centrifugation at 8000× *g* and 4 °C for 10 min, 150 μL of fermentation broth and 50 μL of internal standard (IS, 5 μg/mL 4-amino salicylic acid) were extracted with 500 μL of ethyl acetate. The supernatant was dried under a nitrogen stream after centrifugation at 11,000× *g* for 15 min. Subsequently, the dried residue was mixed with 30 μL of BSTFA (with 1% TMCS) and derivatized at 70 °C for 60 min before analysis with GC-MS. An Agilent 7890A gas chromatography system coupled with an Agilent 5975C inert MSD system was employed to analyze the metabolites. Samples were separated on a film thickness (0.25 μm, 30 m × 0.25 mm i.d), Rxi-5 Sil capillary column (Restek corporation, Bellefonte, PA, USA). The column temperature was initially held at 70 °C for 2 min, and then it was raised to 160 °C at a rate of 6 °C/min, to 240 °C at a rate of 10 °C/min, and to 300 °C at a rate of 20 °C/min, followed by isothermal period of 6 min. The total run time was 34 min. Helium was used as a carrier gas at a flow rate of 1 mL/min. The injector was heated to 250 °C and was on splitless mode, and the injection volume was 1.0 μL. The transfer line, and the electron impact ion source were set at 250 and 230 °C, respectively. The MSD acquired data in the full scan mode (mass range 50–600), with the electron energy of 70 eV. The metabolite content was calculated by Equation (S9).

$$\mathrm{RC} = \frac{A_m}{A_{IS}} \quad \text{(S9)}$$

where RC is the relative content of the metabolite; A_m is the peak area of the metabolite, and A_{IS} is the peak area of internal standard (IS, 5 μg/mL 4-amino salicylic acid).

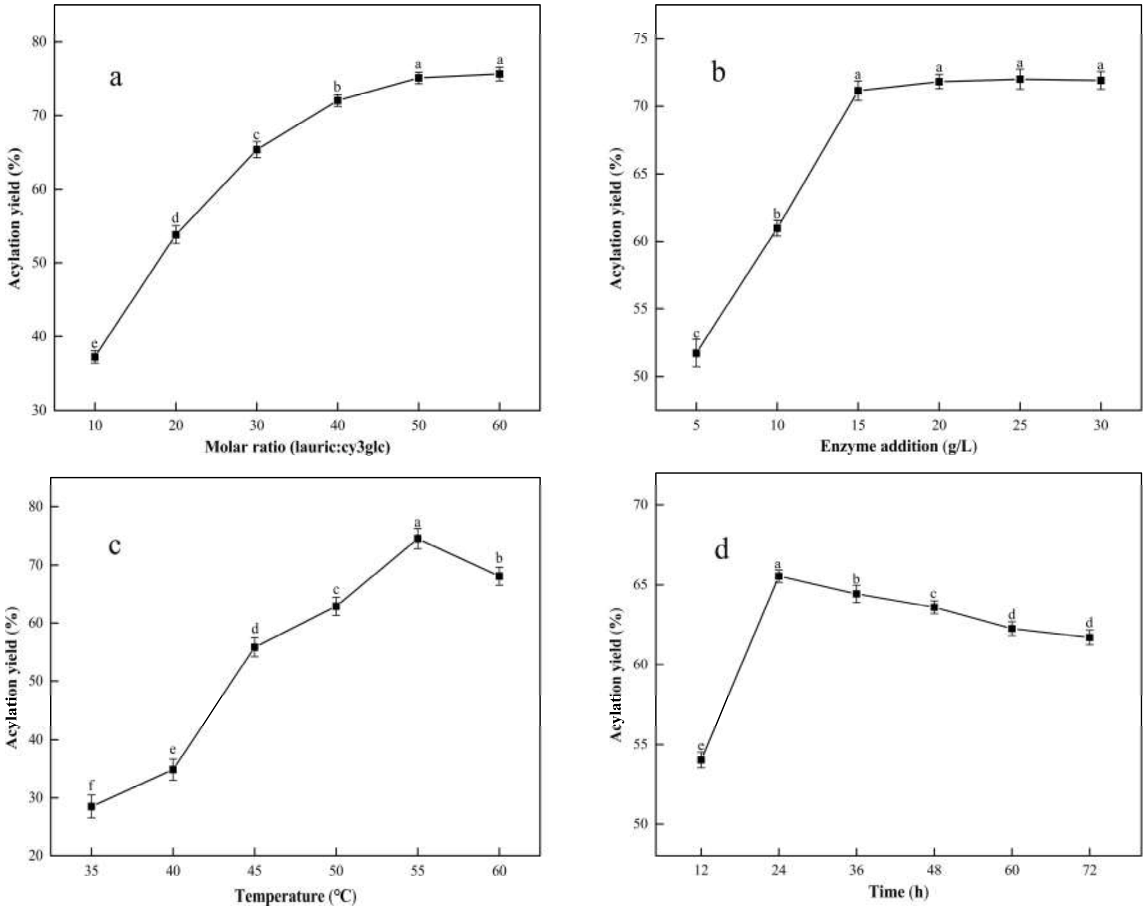

Figure S1. Effect of reaction parameters on acylation yield of Cy3glc. molar ratio (**a**), enzyme addition (**b**), reaction temperature (**c**), and time (**d**).

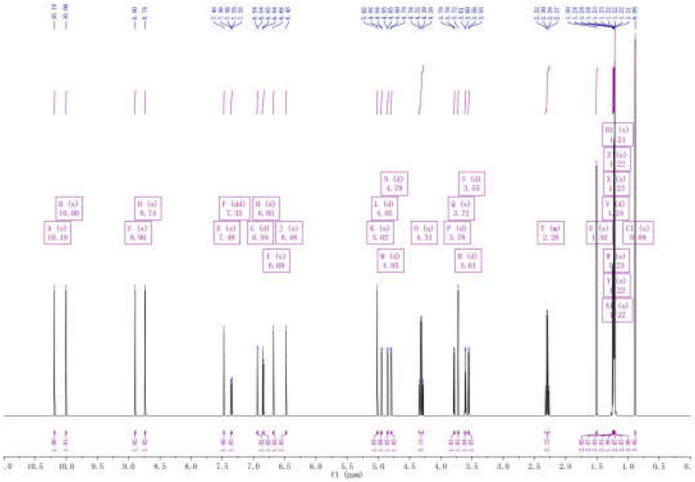

(a) ^{1}H NMR

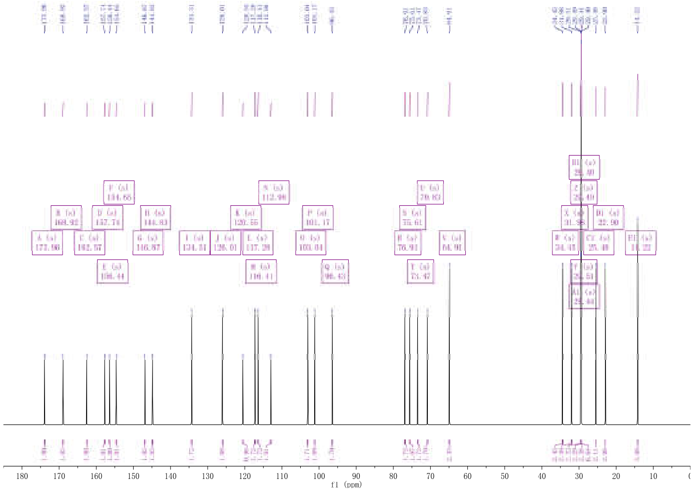

(b) ^{13}C NMR

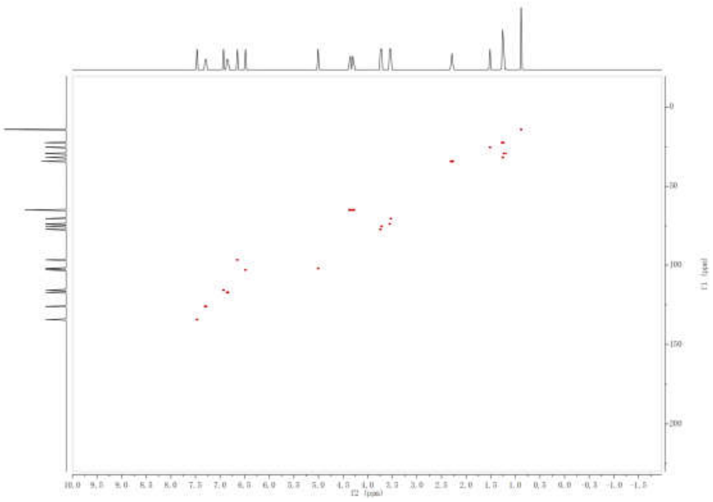

(c) HSQC 2D NMR

Figure S2. ^{1}H, ^{13}C NMR and 2D NMR spectrum (HSQC) for the synthesized compound Cy3glc-C12. (**a**) ^{1}H NMR, (**b**) ^{13}C NMR and (**c**) HSQC 2D NMR.

Table S1. Experimental design, results, and variance analysis of response surface optimization.

(**a**) Coded and actual values of independent variables used for a Box–Behnken design.

**Variable**	Unit	Coded levels		
		–1	0	1
**Molar ratio (X_1)**	/	1:40	1:50	1:60
Enzyme addition (X_2)	g/L	10	15	20
Temperature (X_3)	°C	50	55	60
Time (X_4)	h	12	24	36

(**b**) Experimental data under different reaction parameters for enzymatic acylation of Cy3glc based on a Box–Behnken RSM design (BBD).

**Run**	Variable levels				Responses		**Run**	Variable levels				Responses	
	X_1	X_2	X_3	X_4	Experimental	Predicted		X_1	X_2	X_3	X_4	Experimental	Predicted
**1**	-1	1	0	0	56.38	56.70	16	0	-1	-1	0	60.64	60.46
2	-1	0	0	-1	48.51	48.19	17	0	1	0	1	64.41	63.88
3	0	0	-1	1	66.32	66.93	18	0	-1	0	1	63.95	64.07
4	1	1	0	0	65.49	66.16	19	0	-1	0	-1	50.72	51.56
5	1	0	0	1	66.55	66.56	20	-1	0	0	1	56.71	56.90
6	1	-1	0	0	63.07	62.75	21	0	-1	1	0	61.01	61.22
7	0	0	0	0	64.17	66.83	22	-1	-1	0	0	52.91	52.24
8	-1	0	-1	0	55.11	55.04	23	1	0	1	0	66.94	67.32
9	0	0	0	0	67.31	66.83	24	0	0	1	1	63.60	63.20
10	0	0	0	0	66.51	66.83	25	0	0	0	0	66.46	66.83
11	1	0	0	-1	59.02	58.50	26	0	1	0	-1	59.42	59.62
12	0	0	1	-1	61.45	60.84	27	0	1	1	0	66.81	66.68
13	0	0	0	0	66.74	66.83	28	0	0	-1	-1	52.12	52.52
14	-1	0	1	0	55.54	56.08	29	1	0	-1	0	64.00	63.77
15	0	1	-1	0	63.38	62.85							

(**c**) Analysis of variance (ANOVA) for second polynomial equation for the responses.

**Variable**	***SS***	***DF***	***MS***	***F*-value**	***p*-value**
Model	874.07	14	62.43	172.88	<0.0001**
			linear		
X_1	52.24	1	299.17	828.39	<0.0001**
X_2	62.75	1	46.35	128.34	<0.0001**
X_3	56.70	1	15.80	43.74	<0.0001**
X_4	66.16	1	210.83	583.79	<0.0001**
			Interactions		
X_1X_2	52.52	1	0.27	0.76	0.3993[ns]
X_1X_3	60.84	1	1.57	4.35	0.0557[ns]
X_1X_4	66.93	1	0.11	0.30	0.5916[ns]
X_2X_3	63.20	1	2.35	6.52	0.0230*
X_2X_4	48.19	1	17.02	47.12	<0.0001**
X_3X_4	58.50	1	36.27	100.43	<0.0001**
			Quadratic		
X_1^2	56.90	1	149.92	415.12	<0.0001**
X_2^2	66.56	1	42.49	117.66	<0.0001**
X_3^2	60.46	1	14.02	38.83	<0.0001**
X_4^2	62.85	1	130.68	361.86	<0.0001**
Lack of fit	5.06	14	0.36	3.10	0.1431[ns]
CV%			0.98		
R^2			0.9885		

SS, *DF*, *MS*, and *CV*% represent sum of squares, degree of freedom, mean square, and coefficient of variation, respectively. Highly significant (**), $p < 0.01$; significant (*), $p < 0.05$; not significant (ns), $p > 0.05$.
